# New ex vivo method to objectively assess insulin spatial subcutaneous dispersion through time during pump basal-rate based administration

**DOI:** 10.1038/s41598-023-46993-1

**Published:** 2023-11-16

**Authors:** Pauline Jacquemier, Yann Retory, Clara Virbel-Fleischman, Alexandra Schmidt, Agnes Ostertag, Martine Cohen-Solal, Fawaz Alzaid, Louis Potier, Jean-Baptiste Julla, Jean-François Gautier, Nicolas Venteclef, Jean-Pierre Riveline

**Affiliations:** 1 Institut Necker Enfants Malades (INEM), IMMEDIAB Laboratory, Université de Paris Cité, INSERM U1151, Paris, France; 2 Centre Explor, ALHIST - Air Liquide Healthcare, Bagneux, France; 3 LVL Médical Groupe, Lyon, France; 4 CIAMS, Univ. Paris-Sud, Université Paris-Saclay, 91405 Orsay Cedex, France; 5 CIAMS, Université d’Orléans, 45067 Orléans, France; 6 Université Paris Cité, Inserm U1132 BIOSCAR, 75010 Paris, France; 7 Service de Rhumatologie, Lariboisiere Hospital, AP-HP, 75010 Paris, France; 8 Dasman Diabetes Institute, Kuwait City, Kuwait; 9 Université Paris Cité, UFR de Médecine, Paris, France; 10 Department of Diabetology, Endocrinology and Nutrition, Bichat Hospital, APHP, Paris, France; 11 Sorbonne Université, INSERM, Institut Pierre Louis d’Epidémiologie Et de Santé Publique, 75013 Paris, France; 12 Service of Diabetology, Endocrinology and Nutrition, Federation de Diabetologie, Lariboisiere Hospital, 2 Rue Ambroise Paré, 75010 Paris, AP-HP France

**Keywords:** Type 1 diabetes, Drug delivery

## Abstract

Glycemic variability remains frequent in patients with type 1 diabetes treated with insulin pumps. Heterogeneous spreads of insulin infused by pump in the subcutaneous (SC) tissue are suspected but were barely studied. We propose a new real-time ex-vivo method built by combining high-precision imaging with simultaneous pressure measurements, to obtain a real-time follow-up of insulin subcutaneous propagation. Human skin explants from post-bariatric surgery are imaged in a micro-computed tomography scanner, with optimised parameters to reach one 3D image every 5 min during 3 h of 1UI/h infusion. Pressure inside the tubing is recorded. A new index of dispersion (IoD) is introduced and computed upon the segmented 3D insulin depot per time-step. Infusions were hypodermal in 58.3% among 24 assays, others being intradermal or extradermal. Several minor bubbles and one occlusion were observed. IoD increases with time for all injections. Inter-assay variability is the smallest for hypodermal infusions. Pressure elevations were observed, synchronised with air bubbles arrivals in the tissue. Results encourage the use of this method to compare infusion parameters such as pump model, basal rate, catheter characteristics, infusion site characteristics or patient phenotype.

## Introduction

Patients living with type 1 diabetes are exposed to multiple glycemic disorders, in particular hyperglycemia, hypoglycemia and high glycemic variability (GV)^1,2^. Current innovations focus on increasing time in the target glycemic range, reducing GV, and reducing the number of hypoglycemic events^3^. Among them, insulin pumps or continuous subcutaneous insulin infusion (CSII) are electro-mechanical devices that ensure continuous subcutaneous (SC) delivery of rapid insulin analogs^3,4^. For most patients, CSII largely contribute to reducing GV^5^. CSII are also a major component of closed-loop systems, also sometimes called “artificial pancreas”. These systems, which are nowadays reaching an increasing number of patients and will probably become the reference treatment for patients with type 1 diabetes^6^, rely on a glucose sensor providing continuous glucose monitoring and a control algorithm to adapt CSII delivery through time according to the patient’s specific needs during the day^6^.

However in many patients, a high GV remains frequent despite CSII use, without being satisfactorily explained by sporadic copathologies, patient behaviours such as irregular food intakes, irregular physical activities or poor CSII management.

A heterogeneous diffusion of CSII delivered insulin is an often suspected cause for GV although it was not extensively studied. Some studies provide compelling points in favour of the hypothesis that SC insulin absorption variations impact GV. For instance, Luijf et al. observed higher postprandial glycemic excursions immediately after CSII catheter was changed, and thus a new injection site was being used, compared to after 3 days of use^7^. This observation suggests that SC insulin absorption could vary according to catheter age. Famulla et al. demonstrated impaired insulin absorption and higher intra-patient variability for injections in lipodystrophic areas using both a euglycemic clamp study and mixed-meal tolerance tests^8^. Heinemann and Krinelke even describe infusion sets as the “Achilles heel” of CSII delivery^9^, which is conditioned by a reproducible absorption of insulin in the SC tissue^10^. This leads to the hypothesis that inhomogeneous spread of insulin could occur, and highlights the need to increase knowledge of the way insulin spreads into the tissue for basal rate CSII infusion, and in particular in the few hours following a change in catheter^7^.

Furthermore, beyond catheter age and lipodystrophies^8,10,11^, other factors could alter basal insulin delivery as air bubbles^12^, variations in vasculature, or pressure applied to injection site leading to insufficient SC delivery.

However, although this is the delivery mode in which patients spend the major part of their time, there is currently a lack of method to perform such in depth study of subcutaneous diffusion in basal insulin delivery. Indeed, literature provides multiple examples of bolus delivery studies, either in vivo on swine using imaging^13–15^, in vivo in humans using pressure measurement^16,17^, or ex vivo using imaging^18–20^. All of these develop indispensable tools and indicators evaluating bolus delivery, and have paved the way to studying basal delivery.

The absence of a method to achieve such analysis of basal rate infused insulin can be explained by the extremely low flow rates and volumes associated with those delivery rates. Indeed, despite insulin pumps being called “continuous infusion” systems, they are built to inject by multiple impulses (or strokes)^21,22^. For instance, a 1 unit of insulin per hour (UI/h) rate will be delivered by a Tandem t:slim^®^ pump through 8.3 µL impulses of insulin delivered every 5 min. Volumes of the same order of magnitude would be injected by other manufacturers' insulin pumps. Images of such small infused volumes are challenging to achieve and to our knowledge, not reachable in vivo.

Quantitative description is necessary to enable comparison between CSII infusion parameters in the context of basal delivery. Incidentally, Mader et al.^23^ described in 2013 that an increased surface-to-volume ratio (STVR) of the subcutaneous depot increases insulin absorption as it then involves more capillaries, by using a strategy of dispersed bolus injections. Therefore, the imaging method was chosen to enable such computation for small volume injections such as those delivered at each impulsion of a standard rate of basal delivery. The following method is built on the relevance of a high STVR of the insulin depot for optimal insulin bioavailability.

To fill this gap of method to describe insulin propagation in the tissue for the specific context of basal rate delivery, we propose a descriptive, human sample based, ex vivo method linking basal rate insulin delivery and SC spread of insulin using both continuous measurement of catheter pressure, and repeated high-precision 3D imaging using micro-computed tomography (µCT) scans. We obtained new dynamic indicators to describe insulin propagation in the specific case of CSII basal delivery.

## Results

### Explants infusion sites description

**Figure 1:**
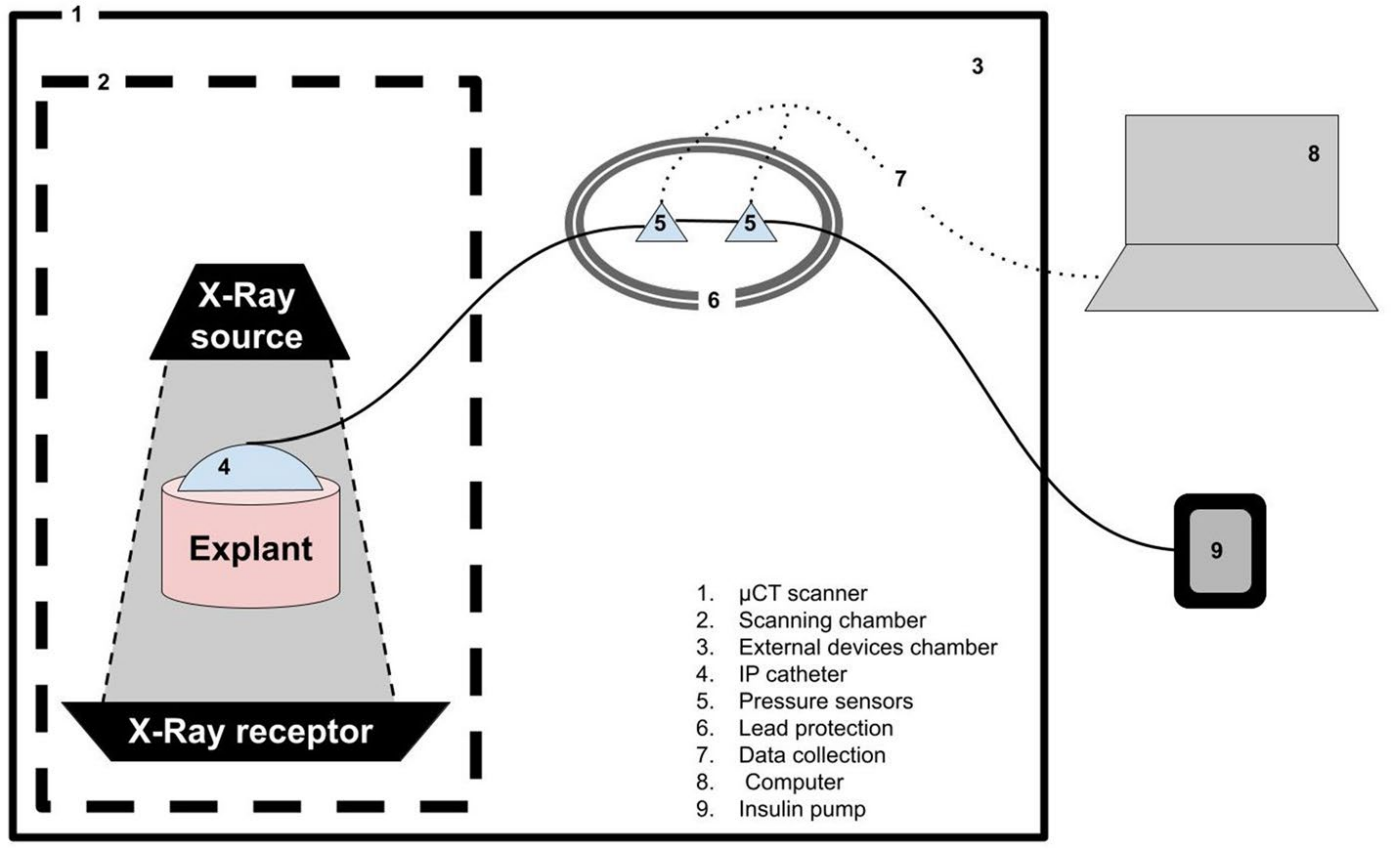
Experimental setting. 1: Micro-CT scanner, 2: Scanning chamber, 3: External devices chamber, 4: CSII catheter, 5: Microfluidic pressure sensors, 6: Lead protection, 7: Data collection wiring, 8: Computer, 9: Insulin pump.

Twenty-four abdominal skin explants were collected from four different human subjects who underwent post-bariatric surgery. They were dimensioned into 3 cm diameter cylinders with a 2 cm thickness so that all three layers of skin tissue (epidermis, dermis, and hypodermis) were present, and infusion catheters were inserted in them. An insulin pump filled with insulin and contrast agent was connected to two microfluidic pressure sensors, and then to the catheter Montage is presented in Fig. 1.

Pump was started 10 min after initiating the acquisition cascade, so that images before infusion were also available for analysis. For this pilot, a fixed basal rate of 1UI/h during 3 h was set. One 3D image was acquired every 5 min during 3 h of infusion.

**Figure 2:**
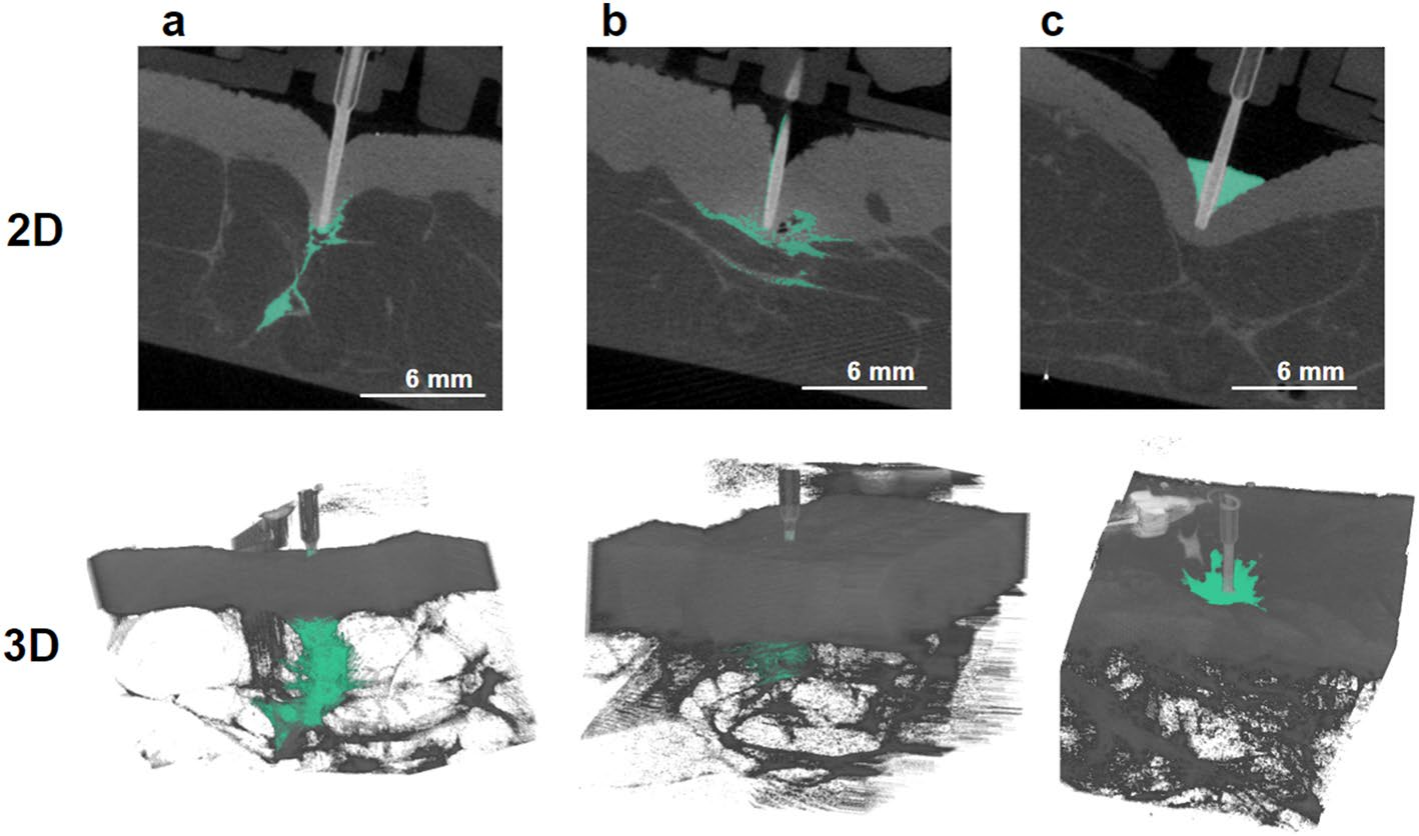
2D and 3D images of infused explants, with infused insulin and contrast agent numerically identified in green. (**a**) hypodermal infusion, (**b**) intradermal infusion, (**c**) extradermal infusion.

Although CSII catheters target the hypodermis, we observed that hypodermis was reached by the cannula in 58.3% (14/24) of the injections, as depicted in Fig. 2a (both in 2D and 3D) where the insulin identified by the segmentation algorithm is numerically coloured in green. The tip of the cannula ended at the limit between dermis and hypodermis in 12.5% (3/24), whereas 20.8% (5/24) of injections were intradermal as in Fig. 2b. For one injection, the cannula did not fully pierce the dermis and the injection was hence extra-dermal (Fig. 2c). For one injection, images were unexploitable due to an acquisition defect.

During all 5 intradermal injections, insulin spread into the dermis, but in 3/5, it also partly spread in the hypodermis. In the 3 injections where the cannula was at the limit between dermis and hypodermis, in two of them insulin was ultimately found only in hypodermis, whereas in one, it spread both in dermis and hypodermis.

### Privileged propagation in interlobular septum in hypodermal injections

**Figure 3:**
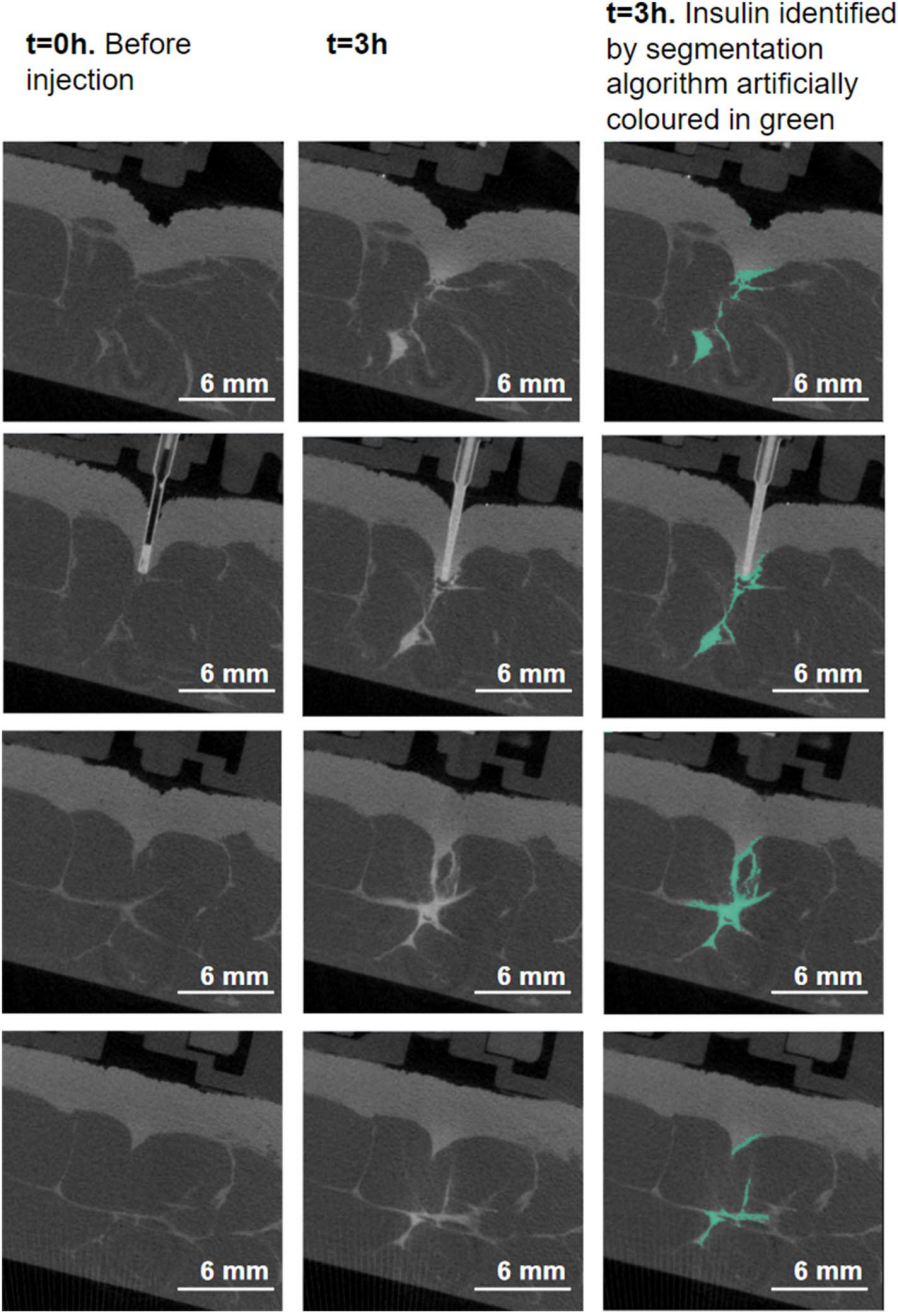
2D slices of one infused explant. Column 1: slices of the explant before infusion was launched. Column 2: corresponding slices after 3 h of insulin infusion. Column 3: corresponding slices after 3 h of infusion, where the insulin and contrast agent mixture detected by the algorithm was numerically identified in green.

Upon all images, the interlobular septum, which divides the subcutaneous tissue into lobules, is clearly visible, as its main component is collagen fibers which are denser than the fat tissue itself. Figure 3 shows extracted slices at various depths in one single explant. The first column is composed of slices extracted from the initial acquisition, when no insulin had yet been delivered. The interlobular walls are visible there.

*.*

On the corresponding slices after 3 h of injection, in the second column, one can see that the white spread of insulin is located along the interlobular walls that had previously been identified. The third column shows which areas of the images were selected by the segmentation algorithm to be insulin and contrast agent (numerically coloured in green).

In the other hypodermal injections, similar visual comparison leads to the same observation: insulin appears to have spread along the interlobular septum.

### Some events leading to under-deliveries

**Figure 4:**
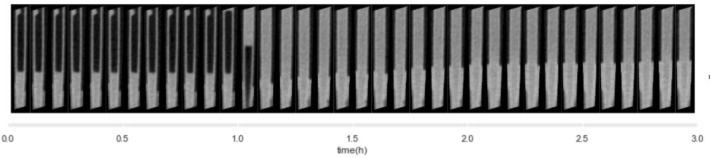
Cannula content during 3 h of infusion, during a test where an occlusion occurred.

Secondly, some under-deliveries were observed: in 18/24 of injections, some minor bubbles were observed at some point in the cannula, leading to the presence of air bubbles imprisoned in the tissue. In one injection, no insulin flow occurred in the cannula during the first hour, as shown by the identical state of the cannula in all of the 13 first 5-min acquisitions. Figure 4 displays the content of the cannula for each 5 min time slot throughout this particular 3 h-test, and in parallel, tubing pressure recorded during that test. A 70 min step-wise pressure elevation reaching 557 mBar was associated with this occlusion. This occlusion did not trigger any pump alarm and also happened to occur during the one extra-dermal injection.

### Introducing a new metric for insulin dispersion: the index of dispersion (IoD)

At each time step, the external surface (S_measured_(t)) and volume (V_measured_(t) of the insulin depot are computed, allowing to generate the surface-to-volume ratio at each time step.

In order for this ratio to be interpretable, the most minimal surface-to-volume ratio mathematically possible for the given volume of injected insulin is also computed: namely, a spherical volume. To this purpose, the radius R_compact_(t) of the sphere which volume V_compact_(t) would be of the same volume as V_measured_(t), is first computed as shown in Eq. (1), leading to the computation of the surface of that theoretical sphere S_compact_(t) in Eq. (2).

This corresponds to the theoretical most compact situation where the insulin depot would have a perfect spherical shape, therefore having a minimal amount of capillaries involved in ensuring absorption into the vascular system. It is the less favourable scenario for insulin absorption.

1 $${R}_{compact}(t)=( {\frac{3 {V}_{measured}\left(t\right)}{4\pi } ) }^\frac{1}{3}$$

2 $$\begin{gathered} Surface_{compact} \left( t \right) = 4\pi R_{compact} \left( t \right)^{2} \hfill \\ Surface_{compact} \left( t \right) = \left( {4\pi } \right)^{\frac{1}{3}} \left( {3V_{measured} \left( t \right)} \right)^{\frac{2}{3}} \hfill \\ \end{gathered}$$

We introduce an index of dispersion IoD(t) which is the surface-to-volume measured ratio ${Surface\_to\_volume\_ratio}_{measured}(t)$ divided by the corresponding spherical surface-to-volume ratio ${Surface\_to\_volume\_ratio}_{compact}(t)$, as visible in Eq. (3). It translates how high the surface of contact between the tissue and the insulin depot is, compared to the most compact situation. This index is expressed without unit. Equation (3) presents a progressive simplification to the initial definition of IoD(t) so to reach a definition that includes our measurable variables, namely ${Surface}_{measured}(t)$ and ${V}_{measured}\left(t\right)$.

3 $$\begin{aligned} IoD\left( t \right)\, & = \frac{{Surface\_to\_volume\_ratio_{measured} \left( t \right)}}{{Surface\_to\_volume\_ratio_{compact} \left( t \right)}} \\ IoD\left( t \right)\, & = \frac{{Surface_{measured} \left( t \right)}}{{Volume_{measured} \left( t \right)}} \frac{{Volume_{compact} \left( t \right)}}{{Surface_{compact} \left( t \right)}} \\ IoD\left( t \right)\, & = \frac{{Surface_{measured} \left( t \right)}}{{Surface_{compact} \left( t \right)}} = \frac{{Surface_{measured} \left( t \right)}}{{\left( {4\pi } \right)^{\frac{1}{3}} \left( {3V_{measured} \left( t \right)} \right)^{\frac{2}{3}} }} \\ \end{aligned}$$

### Index of dispersion (IoD) evolution of the SC insulin through time

**Figure 5:**
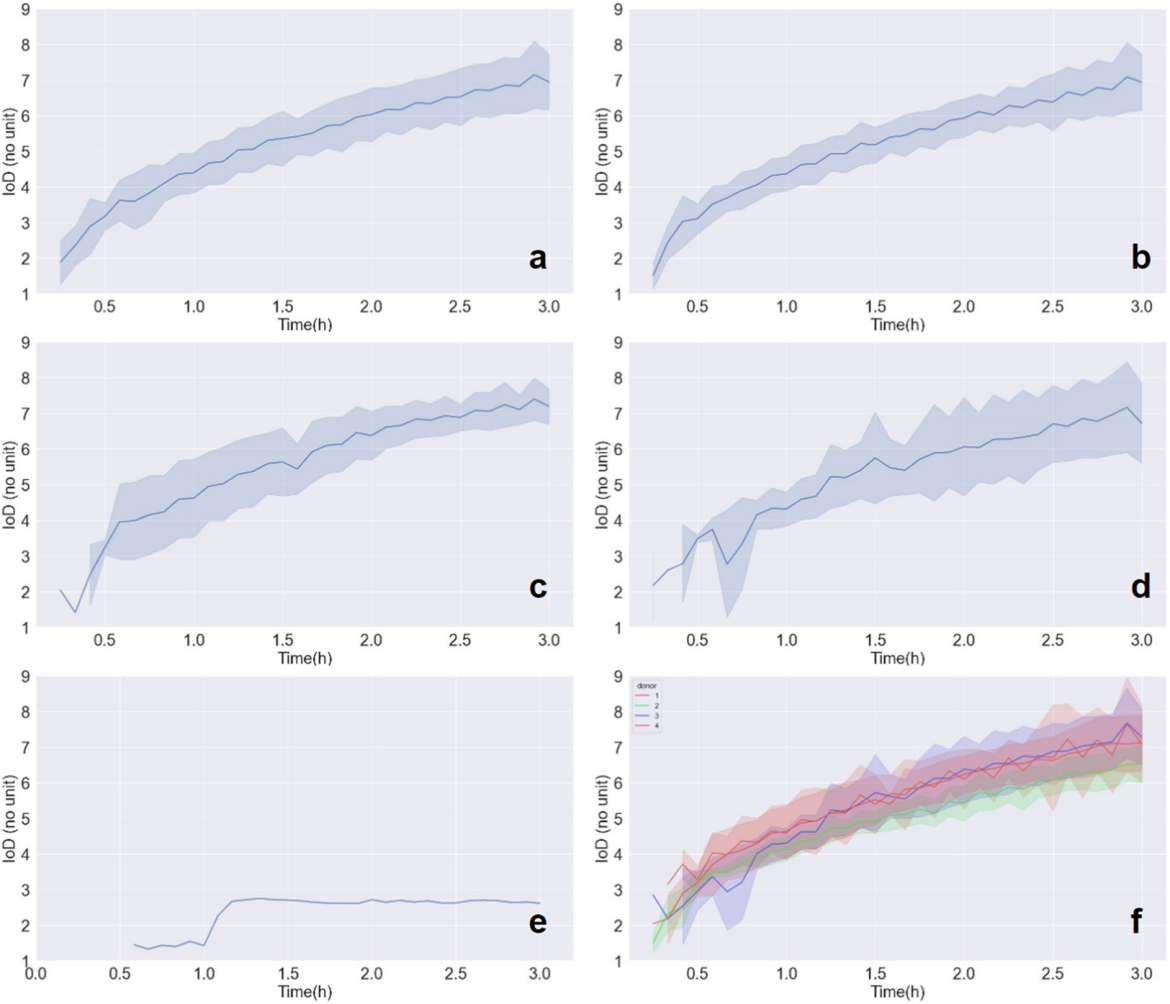
Index of dispersion (IoD) evolution through time, with mean and standard deviation. (**a**) All infusions (N = 21), (**b**) Infusions reaching hypodermis (N = 13), (**c**) Infusions reaching dermis/hypodermis limit (N = 3), (**d**) Infusions reaching dermis (N = 4), (**e**) Extradermal infusion (N = 1), (**f**) All infusions, aggregated by donor (N = 21).

**Figure 6:**
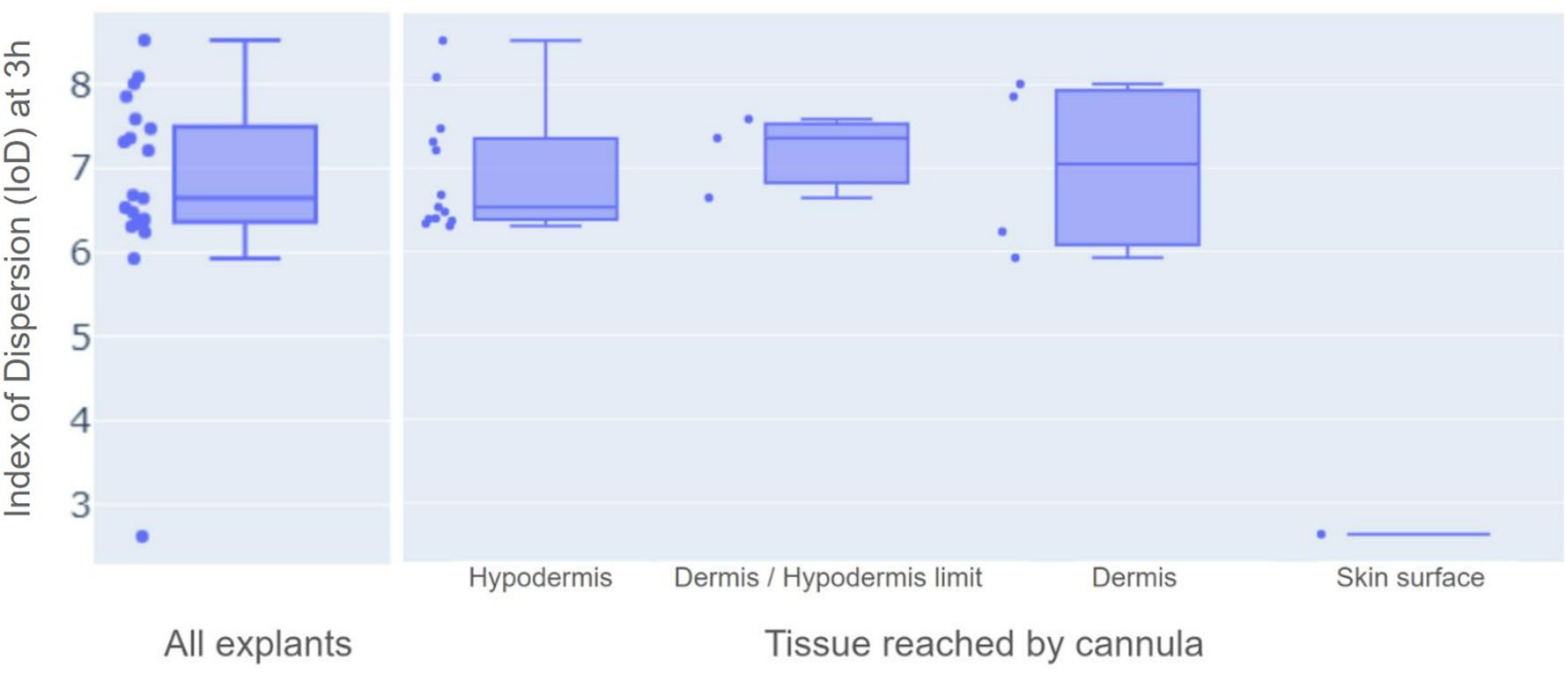
Boxplot of IoD values after 3 h of infusion presenting minimas, maximas, quartiles and median. Classified according to the tissue structure reached by the cannula.

Upon all injections, the IoD increases with time, as depicted in Fig. 5a to d where evolution of mean and standard deviation of IoDs across tests is visible. Throughout all test duration, mean IoDs tend to be comparable from one explant to another and from one patient to another (see Fig. 5f). Values of IoD at 3 h are relevant for it is when the insulin depot is the largest in our tests, and therefore when IoD values are the most precise. They are of importance in the context where this method is used again in future research, in other infusion conditions such as modified basal rate, infusion site or infusion set model. The values of IoD after 3 h of infusion in our test conditions can then be compared with IoD in these new conditions. Here, a mean final IoD value of 6.98 is reached after 3 h of infusion when considering all our tissue samples (Fig. 5a). A closer snapshot at IoD values at 3 h can be found in Fig. 6, with their distribution presented as boxplots classified according to the tissue structure reached by the cannula. The median value of IoD at 3 h, through all our tests, was 6.64. This corresponds to the left boxplot of Fig. 6. In addition, there seems to be no major difference in index of dispersion values when one compares intradermal injections versus hypodermal injections. On the other side, the only heterogeneous value of IoD is of 2.62 at 3 h (Figs. 5e and 6 “skin surface”) and corresponds to the only extra-dermal injection, where insulin accumulated at the skin surface, inside a crater formed around the cannula (Fig. 2c, both in 2D and 3D).

The mean value of standard deviations of IoD computed upon all 5 min intervals was 0.66 when considering all skin-reaching injections. However it was lower (0.55) when only selecting those reaching the hypodermis, than for those reaching the dermis/hypodermis limit (0.62), and the dermal ones (0.83).

When considering explants separately according to the donor as displayed in Fig. 5f, standard deviation values across all tissue structures infused, upon all 5 min intervals, are quite similar to the global standard deviation are respectively 0.68, 0.35, 0.77 and 0.58 for donors 1 to 4.

IoD values were left uncomputed for two explants due to unsolvable 3D tomographic reconstruction issues.

### Simultaneous pressure and image observation in case of bubbles in the cannula

**Figure 7:**
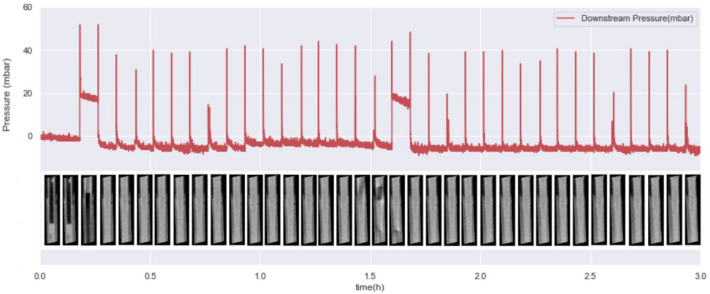
Pressure record and corresponding cannula content for one injection.

As pressure inside the catheter was continuously recorded through the tests, each insulin impulsion was easily identifiable on pressure records (Fig. 7).

Temporary pressure elevations between impulsions were observed, either at the beginning of the test, or at some point during the infusion. In order to understand the potential cause for these elevations, pressure graphs are juxtaposed to the 2D images of the content of the cannula at the given time-step (Fig. 7). We observe that pressure elevations appear to match with the presence of an air bubble inside the cannula the 5 min before the elevation, suggesting that the pressure step matches with the arrival of the air bubble inside the subcutaneous tissue.

## Discussion

This study describes a new method which extends previously existing imaging methods, in order to evaluate the insulin spread in the SC tissue continuously during basal CSII administration. It combines imaging with continuous pressure measurement using two different high precision tools: a µCT scanner whose image acquisition was optimised to acquire one 3D image every 5 min, and continuous pressure sensors. Each 3D image analysis and continuous pressure records were set with the intention to evaluate SC insulin absorption characteristics, and link morphological events with easily pressure-detectable events.

We introduced an objective new indicator to quantify insulin spread, namely the index of dispersion (IoD). It is related to the surface-to-volume ratio (STVR), therefore it increases with STVR, which was shown to be associated with an increased speed of insulin absorption^23^.

Unlike STVR, IoD has the advantage of requiring no comparison with another situation to be interpretable. Indeed, it is built in comparison with the hypothetical case where the insulin depot had a spherical shape, the least favourable situation to diffusion. For instance, an IoD of 7 means that a depot has a surface 7 times higher than the least favourable shape for diffusion. Therefore, one could suppose that the depot has 7 times more probable contacts with capillaries in the SC tissue than it would in the worst-case scenario.

This work is a proof-of-concept aiming to demonstrate feasibility of this method. Therefore, the results demonstrate that IoD, under fixed injection parameters (same pump, same basal rate) and in normal hypodermal infusion, is very reproducible, with a low inter-assay variability (Fig. 5).

Moreover, some interesting results can be highlighted.

The first one is the observation of a higher variability of IoD during ectopic infusion cases, especially when other anatomical structures than hypodermis are infused such as the dermis or the dermis-hypodermis junction. This happens in our model in about 40% of cases. However the number of injections for which the hypodermis was not reached is higher than the proportions found in-vivo by ultrasound detection of the cannula position^11,24^. This is probably due to our ex-vivo insertion method. Indeed, samples are placed inside small Petri dishes of the exact same dimensions as the explants, providing mechanical support to the samples when the inserter hits the skin surface. The use of an inserter does contribute to a more reproducible insertion of the cannula. But the lack of tension at the surface of the epidermis promotes the possible bending of the epidermis surface, thus limiting the depth reached by the cannula tip. Therefore, our experimental model probably participates in explaining this high percentage. Nonetheless, such ectopic cannula positions are at times observed in patients treated with CSII^24^. We hypothesise that it is indeed possible that such events, where non targeted tissue structures are infused, do play a role in some of the glycemic variability observed in patients in daily practice.

Description of such ectopic infusion is then precious data. In particular, despite higher variability in IoD values when the dermis is infused, it is interesting to notice that the mean IoD is not much different in the dermis than in the hypodermis. It was otherwise shown that kinetics of insulin action are different in the dermis and in the hypodermis^25,26^. It is therefore likely that this difference is not caused by a different morphology of the insulin depots infused in the dermis versus in the hypodermis, but rather by a different microvascularization of these two skin structures.

A second interesting observation could be mentioned. As a first method to combine pressure data with imaging data, this study also allows to notice the simultaneity of temporary pressure elevations and air bubbles arrival in the tissue, a quite common situation in real life conditions^12^ leading to under-delivery. This encourages the development of an air bubble detection system based on finer pressure sensors than those already embarked in pumps.

The low value of variability of the IoD throughout experiments and patients in our fixed setting conditions (same pump, same flowrate, same catheter material, length and insertion angle), in hypodermal injection conditions, makes it a reproducible indicator to compare various injection conditions, such as basal rate, CSII model, cannula characteristics, injection site, lipodystrophic tissue, or patient phenotype, in order to better understand SC insulin infusion characteristics.

Our method does present some limitations. Ex-vivo samples do not benefit from any active microvascularization which would contribute to the active transport of insulin from the SC tissue to the bloodstream^27^. Also, the spread of insulin is likely to be improved in the case of perfused tissue compared to ex-vivo tissue due to this active transport. However, a larger insulin spread in the absence of micro-vascularization is likely to imply a larger spread with active micro-vascularization. In that context, IoD measurements in ex-vivo SC insulin administration can still relevantly be used to compare various injection parameters. In the future, in order to address this matter while including insulin clearance due to vascularization, some experimental models that reproduce vascularization could be used^29^. Results generation with such models could allow to model more accurately the active transport of insulin to the blood stream, while including all the variability due to heterogeneous skin characteristics associated with infusion site, patient phenotype or lipodystrophies. Such data could then be relevantly enriching existing in-silico patient simulators^30,31^ which are critical tools in the development of new therapeutic strategies such as automated insulin delivery.

The fact that insulin propagates preferably along the interlobular septum is in accordance with the literature^28,32^. Another limitation must however be acknowledged: our samples come from patients who underwent post-bariatric surgery, and therefore have suffered important weight variations. A known consequence of such weight variations is an irreversible fibrotic adipose tissue^33–36^. Therefore, the interlobular septum of our samples is likely to be thicker and with denser collagen fibres than adipose tissue from patients with type 1 diabetes. Yet such large skin samples from non-obese patients are not easily available for research purposes, for evident ethical reasons. Also, the samples come from female patients only, for during the whole scope of this project, no male tissue was made available to us. This is consistent with the major gender disparity among patients who undergo post-bariatric surgery^37^.

Finally, this work stands as a proof of concept of this evaluation method, so that it can be made available and further used to test the impact of various parameters. In order to do so, it was essential that this study was conducted under fixed conditions of pump, catheter model and basal rate of delivery to assess its reproducibility.

This work, which provides tools to identify the sources of variability in insulin SC absorption caused by heterogeneous diffusion or erratic events, is of importance not only in the context of use of CSII alone, but also as closed-loop systems emerge.

## Methods

### Human skin explants

This ex-vivo study was performed on 24 abdominal skin explants which were collected from surgical residue of post-bariatric surgery for 4 different human subjects (patient data available in supplemental Data S1) after obtaining informed consent from subjects, and in full respect with the Declaration of Helsinki and the article L.1243–4 of the French Public Health Code. BIO-EC Laboratories (Longjumeau, France) possesses an authorization from the Bioethics group of the general director services of the French research and innovation ministry (registered under nDC2008542) to use human skin from surgical waste since 5th May 2010. Explants were dimensioned into 3 cm diameter cylinders with a 2 cm thickness by BIO-EC Laboratories. All 3 layers of skin tissue (epidermis, dermis, hypodermis) were present. From then on, samples were maintained at 37 °C in an incubator (5% of CO_2_, 95% of humidity) with a survival medium (BEMc solution, BIO-EC Laboratories, Longjumeau) renewed every two days until samples were used for experiment.

### Experimental setting description

A Tandem t:slimx2™ insulin pump was connected to two Elveflow™ microfluidic pressure sensors (MPS) manufactured by Elvesys^®^ with ranges of measurement of [− 70 mbar; + 70 mbar] and [− 340 mbar; + 340 mbar], to ensure the most accurate measurement of a broad scale of pressures. Connections were made using tubing from Autosoft 90 catheters. The second MPS was connected to an Autosoft 90 Catheter with a 6 mm Teflon cannula. Montage is presented in Fig. 1.

A 15% mass of Iopamiro 200 mg/mL was mixed with insulin Aspart Novorapid U100 and loaded into a t:slim ™ cartridge. Measurements to quantify the impact of contrast agent on insulin Aspart viscosity are available in supplemental Data S2.

Tubing and catheters were purged with insulin and contrast agent before insertion of the catheter into the tissue sample.

### Radioprotection of the devices

The pump was placed outside of a µCT scanner. The pressure sensors were inside the extended chamber of the µCT, in an area unreachable to X-ray. For full confidence in sensor integrity and measurement, both sensors were surrounded by 3 mm of lead.

### Data acquisition and µCT parameters

µCT acquisitions were obtained using a Skyscan 1176 from Brucker^®^ and parameters were optimised to ensure proper imaging of the sample and the insulin depot while maintaining acquisition time for each 3D image below 5 min. This delay of 5 min was chosen as it constitutes the delay between two impulsions for a Tandem t:slim × 2^22^ and is also to our knowledge the minimum delay between two impulses upon pumps currently on the market.

A cubic region of interest with a 17 mm edge was imaged. Resolution was isotropic, voxel edges were of 17 µm, resulting in a cubic scanned image of 1000 × 1000 × 1000 voxels. The X-Ray source was set at (55 kV, 455 mA) for optimal contrast. A 2 mm Aluminium filter was used in order to limit the beam hardening effect. The rotation step was set at 0.6° and exposure time at 0,240 s.

180° acquisitions were conducted. Upon 3 h of acquisition, one 3D-image per 5 min interval was collected, leading to an overall collection of 36 3D-images. The first image, the third image, and all “uneven” (respectively “even”) 3D images were obtained by clockwise rotation (respectively counterclockwise) of the X-ray source and receptor.

### Image processing

3D tomographic reconstruction was achieved using the NRecon^®^ software (Brucker™). Each slice of the 3D-images were loaded into Matlab^®^ (Mathworks™).

The two first 3D-images were acquired while the pump had not started to deliver, so they could be used as reference of a free-from-insulin tissue. The first image was used as reference for all uneven acquired images, and the second for all even images. Indeed, due to the change in rotation direction between even and uneven images, there was a slight angle tilt between even and uneven images.

On these two first 3D-images, the cylindrical volume corresponding to the inserted cannula in the tissue was manually identified. This corresponding volume was then automatically removed on all following images acquired during the injection.

For each processed 3D-image, the 2D-slices were loaded into the Matlab^®^ environment. The corresponding slices in the free-from-insulin image were subtracted, in order for most of the remaining signal to be the injected insulin and contrast agent mixture. It should be noted that tissue displacement due to the infused volume was minimal, and did not require any image registration step to perform this subtraction. However, should this method be used for larger infused volumes, image registration should be considered before performing the subtraction to ensure alignment of matching tissue structures.

Then, in order to extract insulin depot properties, a linear transformation was performed and a binary image was obtained by using the 0.95 quantile of pixel values as a threshold, allowing to remove noise due to the previous subtraction. Finally, a unique insulin depot object was selected among this binary stack of images by connected-components labeling: only the connected component to include the tip of the cannula (by 18-connectivity) was kept.

The binary objects obtained during this segmentation step were used to compute the external surface and volume of the insulin depot.

## Conclusion

A new evaluation method of SC insulin absorption has been elaborated. To this end, a novel objective indicator for insulin spread was proposed, the index of dispersion, IoD. It is therefore now possible to compare various infusion parameters such as basal rate, CSII model, cannula length, material, infusion site, lipodystrophy, on insulin spread in the SC tissue.

### Supplementary Information

Supplementary Information 1.

Supplementary Information 2.

## Data Availability

The datasets generated during the current study are not publicly available due to the heavy numerical size of the µ-CT 3D images (390 Go of image data for each of the 24 explants) but are available from the corresponding author on reasonable request.
