# Supplementary material for: New ex vivo method to objectively assess insulin spatial subcutaneous dispersion through time during pump basal-rate based administration
Source: Sci Rep. 2023 Nov 16;13:20052. doi: 10.1038/s41598-023-46993-1 (PMC10654403; doi:10.1038/s41598-023-46993-1)
Supplement: Supplementary file 1 — Supplementary Information 1. [file 41598_2023_46993_MOESM1_ESM.docx]

**S1. Patients data**

**Samples provided by Bio-EC laboratories**

All samples were collected on patients who underwent post-bariatric surgery.

|  | **Age** | **Gender** | **Phototype (Fitzpatrick scale)** | **Sampling site** |
| --- | --- | --- | --- | --- |
| **Donor 1** | **55** | **F** | **III** | **abdomen** |
| **Donor 2** | **57** | **F** | **II** | **abdomen** |
| **Donor 3** | **43** | **F** | **V** | **abdomen** |
| **Donor 4** | **51** | **F** | **II** | **abdomen** |
