# Supplementary material for: New ex vivo method to objectively assess insulin spatial subcutaneous dispersion through time during pump basal-rate based administration
Source: Sci Rep. 2023 Nov 16;13:20052. doi: 10.1038/s41598-023-46993-1 (PMC10654403; doi:10.1038/s41598-023-46993-1)

**S2. Viscosity of insulin Aspart Novorapid U100 and Iopamiro 200 contrast agent mixture**

A 15% mass of Iopamiro 200mg/mL was mixed with insulin Aspart Novorapid U100 and loaded into a t:slim ™ cartridge. This concentration results from the comparison between the µCT detectability of a low volume depot for a 10% mass concentration of CA, in accordance with another example in the literature (Mader et al, 2013) and a 15% mass concentration of CA. The increase in viscosity was measured using a Brookfield DV2T viscometer, and found negligible (2% - see further viscosity measurements in figure S1, supplemental data) against the improvement in depot detectability.

| **Concentration CA**  **(% of mass)** | **Viscosity**  **(cP)** | **% increase of viscosity compared to pure Insulin Aspart U100** |
| --- | --- | --- |
| 0 | 1.41 | 0 |
| 5 | 1.42 | 0.71 |
| 10* | 1.47 | 4.26 |
| **15**** | **1.50** | **6.38** |
| 17.5 | 1.53 | 8.51 |
| 20 | 1.57 | 11.35 |
| 22.5 | 1.58 | 12.06 |
| 25 | 1.59 | 12.77 |

* value of CA concentration available in Mader et al, 2013

** value of CA concentration used in this study


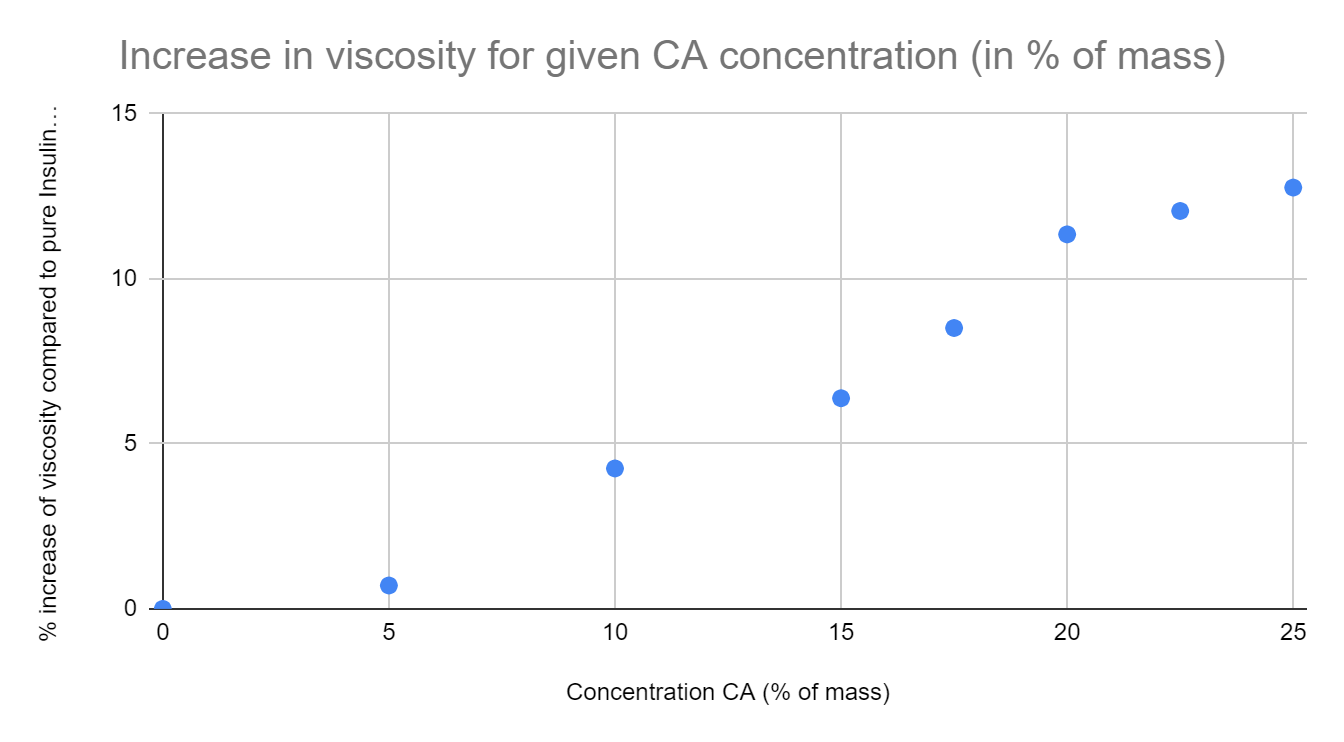

Supplement: Supplementary file 2 — Supplementary Information 2. [file 41598_2023_46993_MOESM2_ESM.docx]
